# Fourfold Filtered Statistical/Computational Approach for the Identification of Imidazole Compounds as HO-1 Inhibitors from Natural Products

**DOI:** 10.3390/md17020113

**Published:** 2019-02-12

**Authors:** Giuseppe Floresta, Emanuele Amata, Davide Gentile, Giuseppe Romeo, Agostino Marrazzo, Valeria Pittalà, Loredana Salerno, Antonio Rescifina

**Affiliations:** 1 Department of Drug Sciences, University of Catania, V.le A. Doria, 95125 Catania, Italy; giuseppe.floresta@unict.it (G.F.); eamata@unict.it (E.A.); davide.gentile@studium.unict.it (D.G.); gromeo@unict.it (G.R.); marrazzo@unict.it (A.M.); vpittala@unict.it (V.P.); 2 Consorzio Interuniversitario Nazionale di ricerca in Metodologie e Processi Innovativi di Sintesi (C.I.N.M.P.S.), Via E. Orabona, 4, 70125 Bari, Italy

**Keywords:** virtual screening, marine products database, natural products database, heme oxygenase, HO-1 inhibitors, imidazole

## Abstract

Over-regulation of Heme oxygenase 1 (HO-1) has been recently identified in many types of human cancer, and in these cases, poor clinical outcomes are normally reported. Indeed, the inhibition of HO-1 is being considered as an anticancer approach. Imidazole scaffold is normally present in most of the classical HO-1 inhibitors and seems indispensable to the inhibitory activity due to its strong interaction with the Fe(II) of the heme group. In this paper, we searched for new potentially HO-1 inhibitors among three different databases: Marine Natural Products (MNP), ZINC Natural Products (ZNP) and Super Natural II (SN2). 484,527 compounds were retrieved from the databases and filtered through four statistical/computational filters (2D descriptors, 2D-QSAR pharmacophoric model, 3D-QSAR pharmacophoric model, and docking). Different imidazole-based compounds were suggested by our methodology to be potentially active in inhibiting the HO-1, and the results have been rationalized by the bioactivity of the filtered molecules reported in the literature.

## 1. Introduction

Heme oxygenase, discovered back in the late sixties, is mostly acknowledged as a family of enzymes deputed to the catabolism of free circulating heme [1]. This reaction produces sole catabolites such as carbon monoxide (CO) and biliverdin (BV) further reduced to bilirubin (BR); both of them endowed with strong cytoprotective activities. It is only in the late eighties that two different isoforms of HO were recognized [2]. The inducible HO-1 isoform (32 kDa protein) is the first characterized and recognized also as heat shock protein 32 (Hsp32). The second isoform is the constitutive HO-2 (36 kDa protein) that is ubiquitously expressed and with abundant levels in the brain and testes. The two proteins share an overall homology of 45% with a peak of 55% in the conserved core regions [3].

**Scheme 1:**
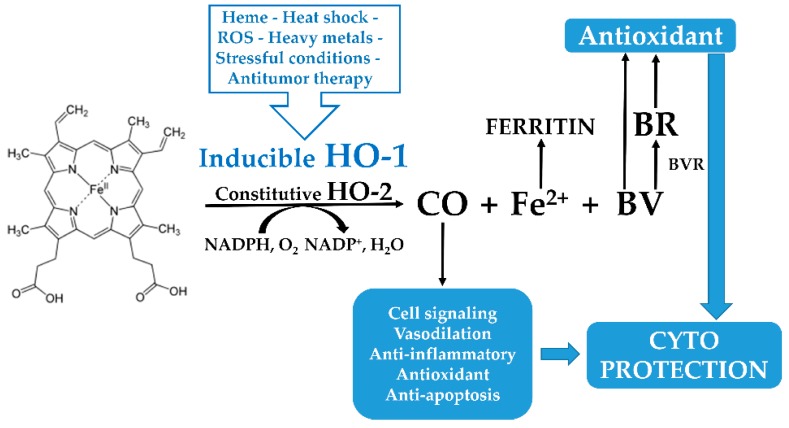
Schematic representation of the heme degradative pathway catalyzed by the heme oxygenase system (HO). HO-2 is a constitutive enzyme, whereas HO-1 is inducible by different noxious stimuli. Elimination of the pro-oxidant heme by both HOs involves cleavage of the protoporphyrin IX ring of heme with production of CO, Fe^2+^, and biliverdin (BV). BV is converted by biliverdin reductase (BVR) into bilirubin (BR). Both CO and BV are bioactive molecules. The Fe^2+^ is then stored by the ferritin. All the products of heme catabolism are cytoprotective. However, overexpression of HO-1 (such as during antitumor therapy) may promote tumor growth and spreading.

HO-1 is mostly expressed in the liver and spleen. In the presence of a diversity of noxious insults, mainly oxidative stress, HO-1 is highly up-regulated. HO-1 expression is mainly transcriptionally regulated by the nuclear factor (erythroid-derived 2)-like 2 (Nrf2), the nuclear factor kappa-light-chain-enhancer of activated B cells (NF-*k*B), and a variety of kinases including protein kinase C (PKC) [4,5]. A crucial role of HO-1 is also the cytoprotection and the preservation of homeostatic equilibrium by modulating inflammatory and antioxidant reactions, regulating cellular proliferation, apoptosis, and immune response. HO-1 overexpression exerts valuable protection in diseases such as lung and cardiovascular injuries, diabetes, metabolic diseases, and transplantation [6,7,8,9,10]. HO-1 induction can be regarded as an evolutionary mechanism of protection that overall safeguards cells and tissues against stressful conditions and stimulates cell-survival. These protective roles are exerted through the enhanced degradation of the pro-oxidant heme and the production of protective by-products CO, BV, and BR (Scheme 1). Nevertheless, it is unlikely that the HO enzymatic activity can mediate such a plethora of protective roles exclusively via its catabolites. Besides the well-known enzymatic activity, relevant signaling functions have been demonstrated for HO-1 and include redistribution of subcellular localization [11]. In fact, even though enzymatically active HO-1 is mainly located in the endoplasmic reticulum, in a truncated and inactive form, HO-1 can move into other compartments such as the nucleus, the caveolae, and the mitochondria [12,13,14]. Migration of HO-1 from the cytoplasm exerts important non-enzymatic signaling functions, resulting in the regulation of gene expression, modulation of protein translation, and activation of DNA repair proteins [11]. Nuclear HO-1 is able to prevent Nrf2 phosphorylation and related ubiquitin-mediated proteolysis, providing Nrf2 accumulation in the nucleus that in turn regulates HO-1 expression itself thus sparking the beginnings of a virtuous circle [15]. Thanks to its cytoprotective roles, HO-1 under physiological conditions inhibits the transition of normal cells to precancerous or malignant ones by preventing ROS-mediated carcinogenesis.

Nevertheless, aberrant levels of HO-1 and Nrf2 have been well-documented in different types of human cancers [16,17,18,19,20]. In fact, by this overexpression, HO-1 could maintain a favorable redox balance in cancer cells by keeping ROS levels in a range that promotes their proliferation and survival, bypass apoptosis, giving rise to tumor growth, metastasis and chemoresistance. In the last decade, there have been dramatic increases in the number of papers that deal with the oncogenic involvement of the HO-1, and cancer patients with HO-1 aberrant overexpression seem to have lower survival rates and poor clinical outcomes. In this context, despite novel chemical inducers of HO-1 are useful in oxidative stress-based diseases [21,22], the identification of novel HO-1 selective inhibitors is of high interest in different types of cancers [23,24]. Other than metalloporphyrins, classical HO-1 inhibitors are characterized by an azole-based structure, with a preference towards a non-substituted imidazole ring, a central linker, and a hydrophobic portion of different size. The imidazole ring is ideal for activity and selectivity towards HO family with respect to other heme-containing enzymes and represents the first anchoring point of the molecule to the Fe^2+^ residue of heme when it is located inside HO-1 heme binding pocket [25]. Specifically, imidazole ring of the inhibitor coordinates with the Fe^2+^ atom of heme substrate through the electron lone pair of nitrogen at the 3-position. This binding prevents the initiation of heme catabolism and results in non-competitive enzymatic inhibition.

Yet, as the imidazole moiety is generally acknowledged for its hepatotoxic activity, its substitution would be desirable [26]. However, any attempt of imidazole substitution performed until today afforded only poor results. The only accepted substitution is with a 1*H*-1,2,4 triazole or a 1*H*-tetrazole ring, but a reduction of the potency of the HO-1 inhibition is generally observed [27]. Consequently, the imidazole nucleus is currently the best choice to design novel HO-1 inhibitors. The only one different scaffold that has been recently described, is present in a small series of quinoline-based imidazole derivatives [28]. Despite the presence of an imidazole nucleus, these molecules represent the only example of HO-1 inhibitors structurally and functionally different from classical imidazole-based HO-1 inhibitors. In fact, dissimilarly to the classical imidazole-based inhibitors, the imidazole ring is substituted at the 2-, 4-, and 5-positions, and these quinoline compounds behave as competitive inhibitors being able to displace the substrate from the catalytic site of the enzyme. More in-depth studies on this class of compounds are necessary to define their importance in the field of HO-1 inhibitors.

*In silico* studies represent a convenient and efficient avenue to the identification of new scaffolds and new bioactive compounds with significant savings of time and money. In agreement with our growing interest in developing selective and potent HO-1 inhibitor and driven by the need of identifying new scaffolds endowed with HO-1 activity and selectivity, we recently reported 2D– and 3D–QSAR models based on the whole collection of HO-1 and HO-2 inhibitors reported so far and collected in a database previously built by our research group (HemeOxDB, http://www.researchdsf.unict.it/hemeoxdb) [29,30,31,32,33,34]. Also, scaffold hopping analysis allowed to design and synthesize new potent HO-1 inhibitors characterized by a novel chemotype obtained by modifying the central region of the ligands [35].

**Scheme 2:**
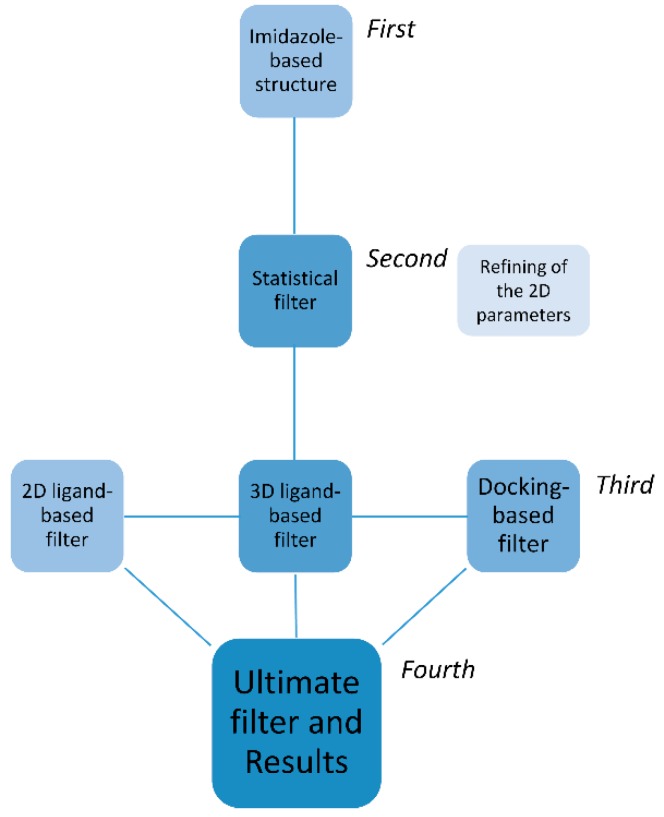
Schematic representation of the used workflow.

As demonstrated, given that *in silico* studies enable the identification of new potent HO-1 inhibitors, in the present paper we report the virtual screening of an imidazole-based moiety fully enriched database obtained by the combination of the three different databases Marine Natural Products (MNP, 14,492 entries), ZINC Natural Products (ZNP, 144,766 entries) and Super Natural II (SN2, 325,319 entries). The entire process was conducted employing a fourfold statistical/computational filtration scheme (Scheme 2).

## 2. Results

### 2.1. First, Second and Third Level of the Statistical/Computational Filtration

**Scheme 3:**
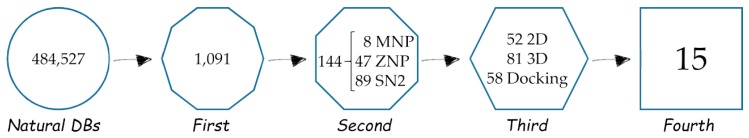
Application of the fourfold filters described in Scheme 2 to the selected natural databases reporting the number of compounds filtered.

The first and second filters used in the selection of compounds were a structural filter and a statistical (based on 2D descriptors) ones. Starting from the three different databases MNP, ZNP, SN2, all the structures containing a non-fused 2-non-substituted imidazole ring were first retrieved, by the substructure filter present in DataWarrior software (5.0.0, Idorsia Pharmaceuticals Ltd, Allschwil, Switzerland) [36], for a total of 1,091 molecules. Then the molecules were filtered through a statistical/2D descriptors filters. To perform this, we analyzed the most potent and selective compounds present in the HemeOxDB [31] retrieving only the molecules presenting an HO-1 IC_50_ value ≤10 μM and an HO-1/HO-2 selectivity ≥10, for a total of 62 entities. The ranges of Molecular weight (200/535), cLogP (–0.35/5.4), cLogS (–5.90/–0.85), H-acceptors (2/8), H-donor (0/1), Druglikeness (–13.20/8.2), DrugScore (0.12/0.96), Total Surface Area (164/390), Relative PSA (0.085/0.35), and Polar Surface Area (18/90) belonging to the 62 potent and selective compounds were all chosen as 2D descriptors and the dataset of 1,091 molecules was further filtered using these interval values to give eight molecules from the MNP, 47 from the ZNP and 89 from the SN2, for a total of 144 molecules (Supplementary Table S1). The number of filtered compounds, for each filter, was reported in Scheme 3.

The selected molecules were then also filtered in a third level using a mixed structure and ligand-based approach. The 2D ligand-based filter is based on an HO-1 inhibitor filter returning for each chemical entity a predicted endpoint expressed as pIC_50_. This 2D-QSAR model was already published [31] and has been built with CORAL software (CORrelation And Logic, version 2016, Istituto di Ricerche Farmacologiche Mario Negri, Milano, Italy) [29,31] employing a Monte Carlo based QSAR analysis [37,38], according to the literature [39,40,41]. Over 144 compounds, 90 have been defined by the model as outliers; this means that the model does not sufficiently describe their 2D chemical structures. The remaining molecules were returned with a predicted endpoint and indicated as falling within the domain applicability. From this subset, 52 compounds have been predicted to possess pIC_50_ values between 2.44 and 7.76.

The same datasets of selected natural products were also evaluated using another ligand-based filter, but this time using 3D descriptors. The 3D molecular structures were aligned to our previous published 3D-QSAR model for the HO-1 receptor [32], and the compounds were then evaluated, as previously reported [42], employing Forge software (v10.4.2, Cresset, New Cambridge House, Hertfordshire, United Kingdom) [43]. Over the whole dataset of natural products, 81 molecules resulted in an excellent or good description by the model. It means that most of the features in the evaluated molecules were well described by the training set of the 3D-QSAR model and the predicted activity can be considered reliable.

The selected molecules were then passed to the structure-based approach adapting the docking procedure already reported for the identification of HO-1 inhibitors [33,34,44]. Of the six HO-1/ligand co-crystallized structures currently published (PDB IDs: 3K4F, 3CZY, 3TGM, 2DY5, 3HOK, and 6EHA) we discarded the 3HOK because the pocket present in the so-called northeastern region, dynamically formed only in this X-ray structure, is not correlated with the potency or selectivity for HO-1 isoform. On the contrary, it has been speculated that this pocket could be correlated with the selectivity for the HO-2 isoform [24,45]. Due to the inherent flexibility of the entire binding site, ranging from 194 Å^3^ (PDB ID 1N45, without ligand) to 585 Å^3^ (PDB ID 3HOK) we choose to use the crystal structure of the HO-1/QC-15 co-crystal (PDB ID 2DY5) in which the cavity is big enough to accommodate different compounds (293 Å^3^) [34]. To validate the docking and considering that in this case it is not possible to use the best docking procedure necessary to correctly accommodate all ligands, i.e., a first roughly docking followed by a Molecular Dynamics and then a re-docking, as reported by us [44], we focalized our attention only to known HO-1 ligands with a potency comparable to that of the co-crystallized QC-15 compound. Therefore, we selected all of the compounds in the HemeOxDB with an experimental HO-1 IC_50_ value ≤5 μM and an HO-1/HO-2 selectivity ≥30 (21 compounds) and docking them to evaluate their *K*_i_. Although a precise correlation does not exist between the experimental measured IC_50_ and the calculated *K*_i_, the obtained results fall into the same values range suggesting a satisfactory predicting capability of the structure-based method. The docking evaluation of our database has highlighted as many as 58 potential new inhibitors with inhibition values in the range p*K*_i_ 6.01–8.09 (1.00–0.01 μM, Supplementary Table S2 green highlighted).

**Figure 1:**
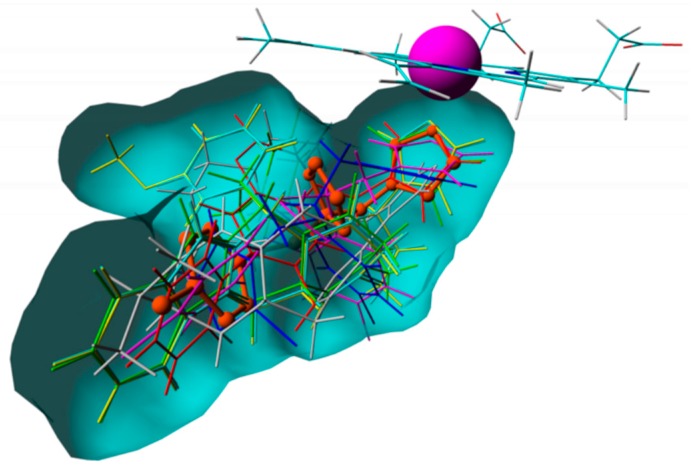
Binding pose of our best compounds (the first seven reported in Table 1) compared with the co-crystallized structure of QC-15 (orange, stick and balls) inside the binding pocket of HO-1 (PDB ID: 2DY5).

All these molecules were selected by the manual inspection of all the docked poses choosing the more reliable, possessing the lowest calculated binding free energy, by means of the classical interaction established by the nitrogen atom of the imidazole ring with the Fe^2+^ atom of the heme moiety, as depicted in the introduction and noticed in all available HO-1/ligand co-crystallized structures; this feature is shown in Figure 1.

### 2.2. The Ultimate Fourth Filter

Finally, to account for all of the obtained results, we further ordered the three datasets according to the mean of the predicted pIC_50_ and p*K*_i_ values obtained by 2D-QSAR, 3D-QSAR, and docking methodologies. The structures of the best 15 compounds have been reported in Table 1. An accurate literature search for the 15 most promising leads, highlighted that three of them could act as inhibitors of heme-containing enzymes. Notably, molecules SN00001674, SN00005909, and SN00213775 have already been reported as CYP3050A1 [46], Heme Oxygenases [47] and sterol 14α-demethylase [48], inhibitors, respectively. It is also worth noting that the calculated average value (6.01, Table 1) of compound SN00005909 (sulconazole) is in excellent agreement with the experimental value (pIC_50_ = 5.96) [47].

**Figure 2:**
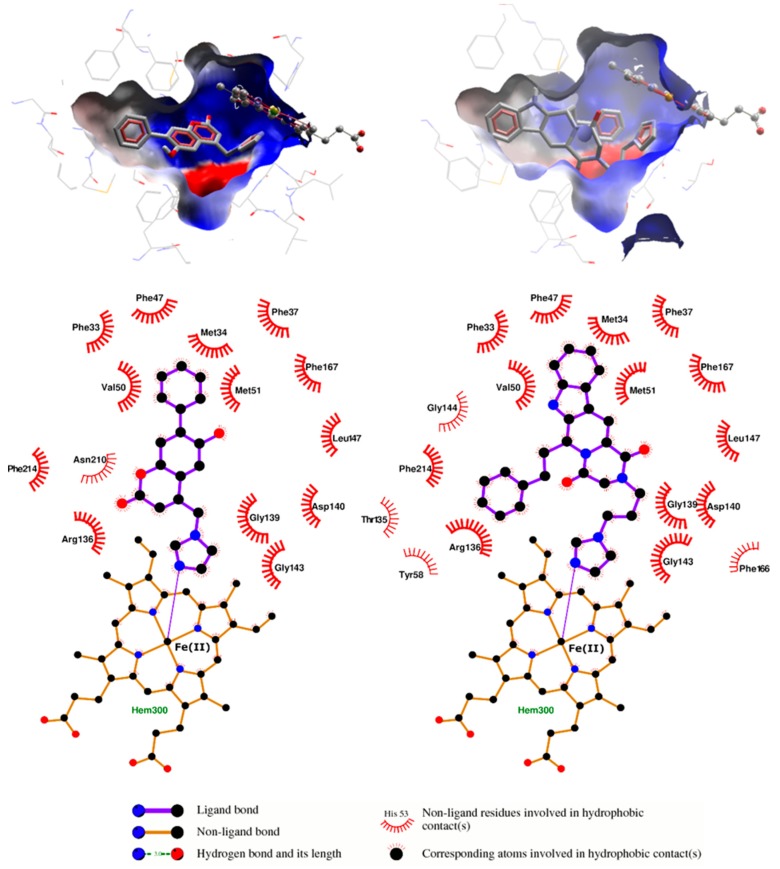
3D-docked poses (upper) of compounds SN00087296 (left) and ZINC08964675 (right) and respective 2D-ligand/protein interaction diagrams (down); *N*-imidazole—Fe(II)-heme interactions are shown as thin violet lines, while the spoked arcs represent residues making nonbonded contacts with the ligand. 2D diagrams were realized with LigPlot+ software (2.1, EMBL-EBI, Wellcome Genome Campus, Hinxton, Cambridgeshire, United Kingdom) [49].

The docked poses of the best two lead compounds SN00087296 and ZINC08964675 reported in Table 1 have been depicted in Figure 2. From their inspection it is evident that the imidazole ring is involved in the interaction with the iron of the heme, as expected for these types of inhibitors.

### 2.3. Very Interesting MNP Out of the Statistical/2D Descriptors Filters

Since none of the eight MNPs residing from the statistical/2D descriptors filters yielded interesting results as an HO-1 inhibitor, we decided to enlarge the mesh considering, of the remaining 342 compounds that had passed the structural filter, those that satisfied both the Lipinski rule of five and at least seven of the statistical/2D descriptors filters. Four of these last compounds have satisfactorily exceeded the remaining filters (2D-QSAR, 3D-QSAR, and docking; i.e., the ultimate fourth filter) and the obtained results are summarized in Table 2 and Table S3.

The first compound of Table 2, oceanapamine [50], is an antimicrobial sesquiterpene alkaloid isolated from the Philippine sponge *Oceanapia* sp., whereas the third compound, verongamine [51], is a histamine H_3_-antagonist isolated from the marine sponge *Verongula gigantea*. Since both HO-1 inhibitors and H_3_-antagonists are implied in the management of the central nervous system (CNS) with diseases such as the Alzheimer [52,53], the verongamine could be a promising starting point to improve the therapeutic management of the latter.

## 3. Materials and Methods

### 3.1. Dataset of Compounds

The chemical structures of the marine and natural dataset were retrieved from Marine Natural Products (MNP, http://docking.umh.es/), ZINC Natural Products (ZNP, http://zinc.docking.org/browse/catalogs/natural-products) and Super Natural II (SN2, http://bioinf-applied.charite.de/supernatural_new/index.php) The full list of the 144 molecules that passed the first filter is available as SMILES for external users in the supplementary material (Table S1).

### 3.2. Structures 2D to 3D Building and Minimization

The structures of all the marine and the natural products were built using Marvin Sketch (18.24, ChemAxon Ltd, Budapest, Hungary) [54]. The 2D structures were first subjected to a molecular mechanics energy minimization by the Merck molecular force field (MMFF94) present in Marvin Sketch. The protonation states the molecules were calculated assuming a neutral pH. Before the alignment for the 3D-QSAR filter and for the docking calculation, the geometry of the obtained molecular mechanics 3D structures were further optimized at semi-empirical level using the parameterized model number 3 (PM3) Hamiltonian as implemented in MOPAC package (MOPAC2016 v. 18.151, Stewart Computational Chemistry, Colorado Springs, CO, USA) [55,56,57].

### 3.3. 2D-QSAR

The software CORAL (CORrelation and Logic, version 2016, Istituto di Ricerche Farmacologiche Mario Negri, Milano, Italy) was used for building the 2D-QSAR model using 382 HO-1 inhibitors, as previously reported [29,31]. The SMILES strings of the compounds evaluated in this work were converted in order to have a SMILES depiction that was equal to that used for generating the original HO-1 inhibitors model. To each SMILES, a random endpoint value was associated in order for the software to compare this value with the predicted one. The following regression was used for the prediction of the endpoints:

$$pIC_{50}=0.0000163\left(\pm 0.0147044\right)+0.0473151\left(\pm 0.0001566\right)\times DCW\left(0.41\right)$$

where DCW is defined as the “descriptor of correlation weights”. The regression for the HO-1 pIC_50_ has been developed in a previously published 2D regression model [31].

### 3.4. Compound Alignment for the 3D-Ligand Based Filter

All the 3D semi-empirical derived structures of the selected marine and natural products were imported into the software Forge (v10.4.2, Cresset, New Cambridge House, Hertfordshire, United Kingdom), for the alignment/evaluation of the dataset in the 3D-QSAR model already published [32]. The filed points of each compound (negative, positive, shape and hydrophobic) were calculated and generated using the XED (extended electron distribution) force field in Forge, then the molecules were aligned with the training set of the QSAR model by a maximum common substructure algorithm using a customized and validated set-up [32,58,59]. All the software’s parameters used for the conformation hunt and alignment have been presented in the supplementary material (Figures S1 and S2). The maximum number of conformations generated for each molecule was set to 500. The root-mean-square deviation of atomic positions cutoff, which is the similarity threshold below which two conformers are assumed identical, was set to 0.5 Å. The gradient cutoff for conformer minimization was set to 0.1 kcal/mol. The energy window was set to 2.5 kcal/mol, and all the conformers with calculated energy outside the selected energy window were discarded.

### 3.5. Molecular Docking

Docking experiments were performed employing AutoDock software (4.2.5.1, The Scripps Research Institute, San Diego, California Jupiter, FL, USA) implemented in YASARA (v. 18.12.7, YASARA Biosciences GmbH, Vienna, Austria) [60,61] using 2DY5 PDB ID of the HO-1 in complex with QC-15. The maps were generated by the program AutoGrid (4.2.5.1, The Scripps Research Institute, San Diego, California Jupiter, FL, USA) with a spacing of 0.375 Å and dimensions that encompass all atoms extending 5 Å from the surface of the structure of the crystallized ligand. All the parameters were inserted at their default settings. In the docking tab, the macromolecule and ligand are selected, and GA parameters are set as ga_runs = 100, ga_pop_size = 150, ga_num_evals = 25000000, ga_num_generations = 27000, ga_elitism = 1, ga_mutation_rate = 0.02, ga_crossover_rate = 0.8, ga_crossover_mode = two points, ga_cauchy_alpha = 0.0, ga_cauchy_beta = 1.0, number of generations for picking worst individual = 10.

## 4. Conclusions

In this study, we describe the screening of new potential HO-1 inhibitors reported in a database of 484,577 imidazoles like compounds retrieved from three different databases: Marine Natural Products (MNP), ZINC Natural Products (ZNP) and Super Natural II (SN2). Through a fourfold statistical/computational filtration approach based on 2D descriptors, 2D-QSAR and 3D-QSAR statistical models and docking calculations, different, potentially active imidazole compounds were selected. Among the filtered final compounds some of them demonstrate the capability of heme-containing enzymes inhibition. Interestingly, some of the selected compounds have already been used as an antifungal medication and are already FDA approved drugs, particularly tioconazole and sulconazole. The other two, oceanapamine and verongamine, present antibacterial and histamine H_3_-antagonist activity, respectively. Thus, verongamine, presenting an experimental proved H_3_-antagonist activity and a potential calculated HO-1 inhibitor activity, could act as a double targeting compound to improve the therapeutic management of the Alzheimer and related CNS diseases. The extension of our findings to the complete list of hit compounds in Table 1 and Table 2 can be an avenue worth purchasing for the discovery of novel potent inhibitors of HO-1. Moreover, the natural target of the tioconazole and sulconazole has recently been investigated as off-label in the anticancer chemotherapy [62], and a dual inhibition with HO-1 can be of interest for the field.

## Figures and Tables

**Table 1:** ID, structure, and mean of predicted inhibitory values for the 15 best promising new leads.

ID	Structure	Mean Values (pIC_50_ p*K*_i_ Mixed)
SN00087296	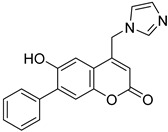	6.49
ZINC08964675	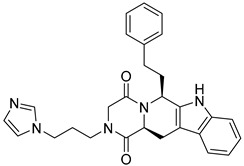	6.29
SN00001674 (tioconazole)	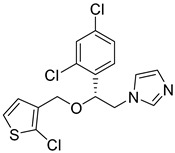	6.25
ZINC08918535	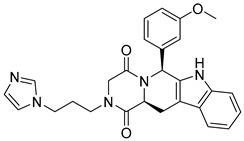	6.14
ZINC08964671	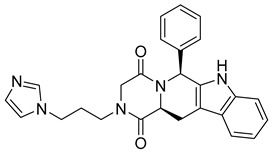	6.02
SN00005909 (sulconazole)	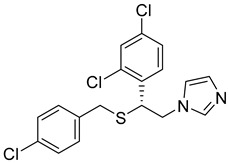	6.01
SN00032231	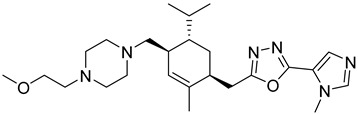	5.98
SN00238920	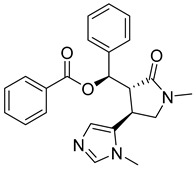	5.87
ZINC03985121	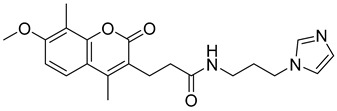	5.85
ZINC08917761	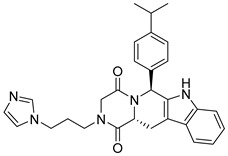	5.83
ZINC03985168	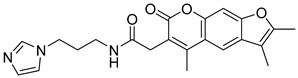	5.78
SN00230416	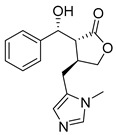	5.71
SN00032287	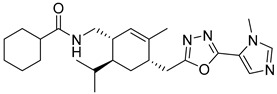	5.70
SN00213775	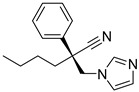	5.67
ZINC03985184	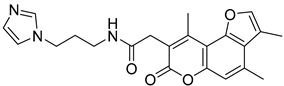	5.65

**Table 2:** ID, structure, and mean of predicted inhibitory values for the four best MNP promising new leads that not strictly satisfied all statistical/2D descriptors filters.

ID	Structure	Mean Values (pIC_50_ p*K*_i_ Mixed)
MNP4580 (oceanapamine)	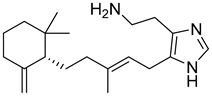	6.31
MNP6510	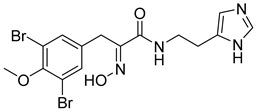	6.04
MNP3690 (verongamine)	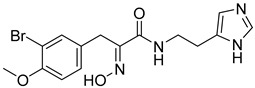	5.94
MNP10136	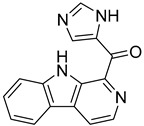	5.58

## Supplementary Materials

The following are available online at https://www.mdpi.com/1660-3397/17/2/113/s1, Figure S1: Forge’s parameters used for the conformation hunt. Figure S2: Forge’s parameters used for the alignment. Table S1: Dataset of filtered natural products containing a non-fused 2-non-substituted imidazole nucleus. Table S2: Calculated values of pIC_50_ (2D and 3D-QSAR) and *K*_i_ (docking) and their mean. Table S3: Calculated values of pIC50 (2D and 3D-QSAR) and Ki (docking) and their mean for outsider marine compounds.
