# Supplementary material for: Fourfold Filtered Statistical/Computational Approach for the Identification of Imidazole Compounds as HO-1 Inhibitors from Natural Products
Source: Mar Drugs. 2019 Feb 12;17(2):113. doi: 10.3390/md17020113 (PMC6409521; doi:10.3390/md17020113)
Supplement: Supplementary file 1 [file marinedrugs-17-00113-s001.pdf]

marine drugs

MDPI

Supplementary Materials

Fourfold Filtered Statistical/Computational Approach for the Identification of Imidazole Compounds as HO-1 Inhibitors from Nat70125ural Products

Giuseppe Floresta [1], Emanuele Amata [1], Davide Gentile [1], Giuseppe Romeo [1], Agostino Marrazzo [1], Valeria Pittalà [1], Loredana Salerno [1,*] and Antonio Rescifina [1,2,*]

[1] Department of Drug Sciences, University of Catania, V.le A. Doria, 95125 Catania, Italy; giuseppe.floresta@unict.it (G.F.); eamata@unict.it (E.A.); davide.gentile@studium.unict.it (D.G.); gromeo@unict.it (G.R.); marrazzo@unict.it (A.M.); vpittala@unict.it (V.P.)

[2] Consorzio Interuniversitario Nazionale di ricerca in Metodologie e Processi Innovativi di Sintesi (C.I.N.M.P.S.), Via E. Orabona, 4, Bari, 70125, Italy

* Correspondence: lsalerno@unict.it (L.S.); arescifina@unict.it (A.R.); Tel.: +39-095-738-5017 (A.R.)

Table of content

Figure S1. Forge's parameters used for the conformation hunt S2

Figure S2. Forge's parameters used for the alignment S2

Table S1. Dataset of filtered natural products containing a non-fused 2-non-substituted imidazole nucleus S3

Table S2. Calculated values of pIC_{50} (2D and 3D-QSAR) and K_i (docking) and their mean S6

Table S3. Calculated values of pIC_{50} (2D and 3D-QSAR) and K_i (docking) and their mean for outsider marine compounds S9

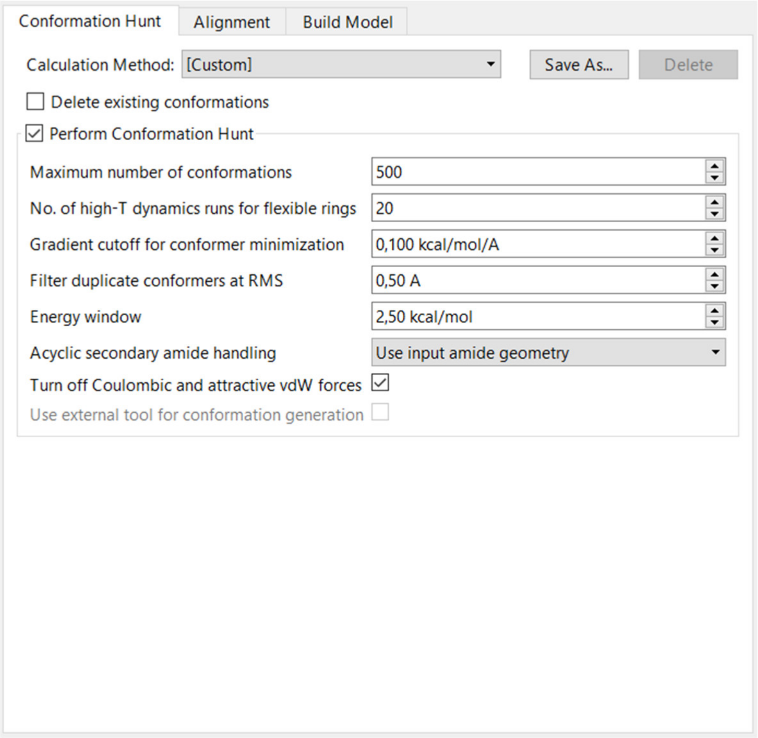

Figure S1. Forge's parameters used for the conformation hunt.

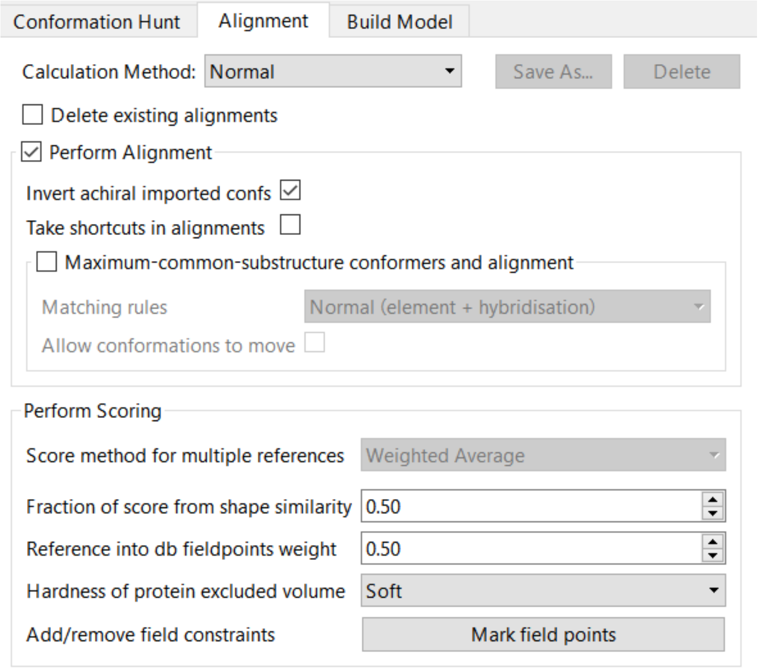

Figure S2. Forge's parameters used for the alignment.

Table S1. Dataset of filtered natural products containing a non-fused 2-non-substituted imidazole nucleus.

ID	SMILES
MNP2902	O(C(=O)\C=C\c1n(cnc1CC=C(C)C)C)C
MNP3090	O(C(=O)C=CC=1[N+](C)(C)[C-]=NC=1CC=C(C)C)C
MNP4640	O=C(C1=NCCc2c1[nH]c1c2cccc1)c1n(cnc1)C
MNP4778	Brc1cc2n(c3c(c2cc1)ccnc3)Cc1nc[nH]c1SC
MNP4779	Brc1cc2n(c3c(c2cc1)ccnc3)Cc1nc[nH]c1S(=O)C
MNP4780	S(C)c1[nH]cnc1Cn1c2c(c3c1cncc3)cccc2
MNP4781	S(=O)(C)c1[nH]cnc1Cn1c2c(c3c1cncc3)cccc2
MNP5523	O(C(=O)\C=C/c1ncn(C)c1CC=C(C)C)C
SN00000116	O(C(=O)c1n(cnc1)[C@@H](C)c1ccccc1)CC
SN00001674	Clc1cc(Cl)ccc1[C@@H](OCc1ccsc1Cl)Cn1cc[nH+]c1
SN00005909	Clc1cc(Cl)ccc1[C@@H](SCc1ccc(Cl)cc1)Cn1ccnc1
SN00021256	O(C(=O)\C=C\c1ncn(C)c1CC=C(C)C)C
SN00031534	o1nc(nc1[C@H]1[NH+](CCC1)Cc1ccc(OC)cc1)-c1ccc(nc1)-n1ccnc1
SN00031535	o1nc(nc1[C@H]1[NH+](CCC1)Cc1ccccc1)-c1ccc(nc1)-n1ccnc1
SN00031537	o1nc(nc1[C@@H]1[NH+](CCC1)Cc1cc2c(nc1)cccc2)-c1ccc(nc1)-n1ccnc1
SN00031538	Clc1ccc(cc1)C[NH+]1CCC[C@H]1c1onc(n1)-c1ccc(nc1)-n1ccnc1
SN00031540	o1nc(nc1[C@@H]1[NH+](CCC1)C1CCCCC1)-c1ccc(nc1)-n1ccnc1
SN00031541	Fc1ccccc1C[NH+]1CCC[C@@H]1c1onc(n1)-c1ccc(nc1)-n1ccnc1
SN00031542	o1nc(nc1[C@H]1[NH+](CCC1)C)-c1ccc(nc1)-n1ccnc1
SN00031543	o1cccc1C[NH+]1CCC[C@H]1c1onc(n1)-c1ccc(nc1)-n1ccnc1
SN00031546	o1nc(nc1[C@@H]1[NH+](CCC1)CC(C)C)-c1ccc(nc1)-n1ccnc1
SN00031547	o1nc(nc1[C@H]1[NH+](CCC1)Cc1ccccc1OC)-c1ccc(nc1)-n1ccnc1
SN00031548	o1nc(nc1[C@@H]1[NH+](CCC1)C1CC[NH+](CC1)C)-c1ccc(nc1)-n1ccnc1
SN00031550	o1nc(nc1[C@H]1[NH+](CCC1)CC(C)(C)C)-c1ccc(nc1)-n1ccnc1
SN00031551	o1nc(nc1[C@@H]1[NH+](CCC1)Cc1cccnc1)-c1ccc(nc1)-n1ccnc1
SN00031552	o1nc(nc1[C@H]1[NH+](CCC1)Cc1ccncc1)-c1ccc(nc1)-n1ccnc1
SN00031553	o1nc(nc1[C@@H]1[NH+](CCC1)C1CCOCC1)-c1ccc(nc1)-n1ccnc1
SN00031555	o1nc(nc1[C@H]1[NH+](CCC1)Cc1ccc(cc1)C)-c1ccc(nc1)-n1ccnc1
SN00031557	o1nc(nc1[C@@H]1[NH+](CCC1)C(C)C)-c1ccc(nc1)-n1ccnc1
SN00031559	o1nc(nc1[C@H]1[NH+](CCC1)Cc1c2c(n(c1)C)cccc2)-c1ccc(nc1)-n1ccnc1
SN00031561	o1nc(nc1[C@@H]1[NH+](CCC1)CC1CC1)-c1ccc(nc1)-n1ccnc1
SN00031563	o1nc(nc1[C@@H]1[NH+](CCC1)C1CCC1)-c1ccc(nc1)-n1ccnc1
SN00031564	o1nc(nc1[C@H]1[NH2+]CCC1)-c1ccc(nc1)-n1ccnc1
SN00032199	o1c(nnc1C[C@@H]1C[C@@H](C(C)C)[C@@H](C=C1C)C[NH+]1CCN(CC1)C(=O)C)-c1n(cnc1)C
SN00032201	o1c(nnc1C[C@@H]1C[C@@H](C(C)C)[C@@H](C=C1C)C[NH2+]CCN1CCOCC1)-c1n(cnc1)C
SN00032203	Clc1ccc(cc1)C[NH2+]C[C@@H]1C=C(C)[C@@H](C[C@H]1C(C)C)Cc1oc(nn1)-c1n(cnc1)C
SN00032204	o1c(nnc1C[C@@H]1C[C@@H](C(C)C)[C@@H](C=C1C)C[NH2+]C1CCCC1)-c1n(cnc1)C
SN00032205	o1c(nnc1C[C@@H]1C[C@@H](C(C)C)[C@@H](C=C1C)C[NH2+]C1CC1)-c1n(cnc1)C
SN00032206	Fc1ccc(cc1)C[NH2+]C[C@@H]1C=C(C)[C@@H](C[C@H]1C(C)C)Cc1oc(nn1)-c1n(cnc1)C
SN00032209	o1c(nnc1C[C@@H]1C[C@@H](C(C)C)[C@@H](C=C1C)C[NH2+]C(C)C)-c1n(cnc1)C
SN00032210	o1c(nnc1C[C@@H]1C[C@@H](C(C)C)[C@@H](C=C1C)C[NH2+]CCOC)-c1n(cnc1)C
SN00032211	o1c(nnc1C[C@@H]1C[C@@H](C(C)C)[C@@H](C=C1C)C[NH2+]Cc1ccccc1OC)-c1n(cnc1)C

SN00032212	o1c(nnc1C[C@@H]1C[C@@H](C(C)C)[C@@H](C=C1C)C[NH+]1CCC[C@@H]1COC)-c1n(cnc1)C
SN00032215	o1c(nnc1C[C@H]1C[C@H](C(C)C)[C@H](C=C1C)C[NH+](CCC#N)C)-c1n(cnc1)C
SN00032216	o1c(nnc1C[C@@H]1C[C@@H](C(C)C)[C@@H](C=C1C)C[NH+]1CCN(CC1)C)-c1n(cnc1)C
SN00032217	o1c(nnc1C[C@@H]1C[C@@H](C(C)C)[C@@H](C=C1C)C[NH+]1CCOCC1)-c1n(cnc1)C
SN00032219	o1c(nnc1C[C@@H]1C[C@@H](C(C)C)[C@@H](C=C1C)C[NH2+]Cc1cccnc1)-c1n(cnc1)C
SN00032220	o1c(nnc1C[C@@H]1C[C@@H](C(C)C)[C@@H](C=C1C)C[NH2+]Cc1ccncc1)-c1n(cnc1)C
SN00032221	o1c(nnc1C[C@@H]1C[C@@H](C(C)C)[C@@H](C=C1C)C[NH+]1CCCCC1)-c1n(cnc1)C
SN00032224	o1c(nnc1C[C@@H]1C[C@@H](C(C)C)[C@@H](C=C1C)C[NH+]1CCCC1)-c1n(cnc1)C
SN00032225	o1c(nnc1C[C@@H]1C[C@@H](C(C)C)[C@@H](C=C1C)C[NH+](CCN(C)C)C)-c1n(cnc1)C
SN00032229	o1c(nnc1C[C@@H]1C[C@@H](C(C)C)[C@@H](C=C1C)C[NH+](Cc1cccnc1)C)-c1n(cnc1)C
SN00032231	o1c(nnc1C[C@@H]1C[C@@H](C(C)C)[C@@H](C=C1C)C[NH+]1CCN(CC1)CCOC)-c1n(cnc1)C
SN00032256	o1c(nnc1C[C@@H]1C[C@@H](C(C)C)[C@@H](C=C1C)CO)-c1n(cnc1)C
SN00032284	o1c(nnc1C[C@@H]1C[C@@H](C(C)C)[C@@H](C=C1C)CNC(=O)C)-c1n(cnc1)C
SN00032287	o1c(nnc1C[C@@H]1C[C@@H](C(C)C)[C@@H](C=C1C)CNC(=O)C1CCCCC1)-c1n(cnc1)C
SN00032288	o1c(nnc1C[C@@H]1C[C@@H](C(C)C)[C@@H](C=C1C)CNC(=O)C1CC1)-c1n(cnc1)C
SN00032289	Fc1ccc(cc1)C(=O)NC[C@@H]1C=C(C)[C@@H](C[C@H]1C(C)C)Cc1oc(nn1)-c1n(cnc1)C
SN00065009	ClC1=C(n2ccnc2)C(OC)(OC)[C@@](Cl)(CC=C)C1=O
SN00065121	O=C1N(CCCn2ccnc2)C(=O)N[C@H]1[C@H](CC)C
SN00076641	O1C(=O)C(CCC(=O)n2ccnc2)=C(C2=CC=3C(O[C@@H](C)C=3C)=C[C@-]12)C
SN00086779	O=C(NCCCn1ccnc1)[C@H](CCC)C
SN00087288	O=C1N(CCCn2ccnc2)C(=O)N[C@H]1[C@@H](CC)C
SN00087296	O1c2c(cc(O)c(c2)-c2ccccc2)C(=CC1=O)Cn1ccnc1
SN00087300	O1c2c(cc(cc2)CC)C(=CC1=O)Cn1ccnc1
SN00087305	O1c2c(C(C)=C(CCC(=O)n3ccnc3)C1=O)c(OC)cc1OC(CCc12)(C)C
SN00213775	n1ccn(c1)C[C@](CCCC)(C#N)c1ccccc1
SN00214383	Fc1ccc(cc1)-c1ncn(CC2CC2)c1-c1nc(ncc1)N
SN00230416	O1C[C@@H](Cc2n(cnc2)C)[C@H]([C@@H](O)c2ccccc2)C1=O
SN00238920	O=C1N(C[C@H]([C@@H]1[C@@H](OC(=O)c1ccccc1)c1ccccc1)c1n(cnc1)C)C
SN00243990	O=C/1NC[C@@H](\C\1=C/c1ccccc1)c1ncn(c1)C
SN00253274	O=C(CC(n1cc(nc1)\C=C\C(=O)[O-])(C)C)C
SN00257780	O=C1N(C[C@H]([C@@H]1[C@@H](OC(=O)c1ccccc1)c1ccccc1)c1ncn(c1)C)C
SN00261841	O=C(NCCc1n(cnc1)C)\C=C\c1ccccc1
SN00264937	O=C1N(c2ccccc2CCC(=O)c2n(cnc2)C)C(=O)CC1
SN00265125	O1[C@@]2(OC)C=C[C@]1(C)[C@H](OC(=O)\C=C\c1ncn(c1)C)C[C@H]1[C@@H](C=C2C)[C@@H](CC=C1C)C(C)C
SN00278994	O1[C@@]2(O)C=C[C@]1(C)[C@H](OC(=O)\C=C\c1ncn(c1)C)C[C@H]1[C@@H](C=C2C)[C@@H](CC=C1C)C(C)C
SN00280641	O=C1N(C[C@@H]([C@@H]1[C@H](O)c1ccccc1)c1ncn(c1)C)C
SN00280678	O=C1N([C@@H](O)CC1)c1ccccc1CCC(=O)c1ncn(c1)C
SN00284317	O1C[C@H](Cc2n(cnc2)C)[C@H]([C@@H](O)c2ccccc2)C1=O
SN00286074	O(C(=O)[C@@H]([C@H]1[C@@H](CN(C)C1=O)c1ncn(c1)C)c1ccccc1)c1ccccc1
SN00292230	O=C1N(C[C@@H]([C@@H]1[C@H](O)c1ccccc1)c1n(cnc1)C)C
SN00306630	O=C1N(C[C@H]([C@@H]1[C@@H](O)c1ccccc1)c1ncn(c1)C)C
SN00320806	O1C[C@@H](Cc2n(cnc2)C)[C@H]([C@H](O)c2ccccc2)C1=O
SN00325795	O=C(CCCCCC)CCC(=O)N(CCc1n(cnc1)C(=O)C)C

SN00335204	O=C/1NC[C@@H](\C\1=C\c1ccccc1)c1n(cnc1)C
SN00343943	O=C1N(C[C@H]([C@@H]1[C@H](O)c1ccccc1)c1ncn(c1)C)C
SN00344415	O1C[C@H](Cc2n(cnc2)C)[C@H]([C@H](O)c2ccccc2)C1=O
SN00352003	O(C)c1ccccc1C[C@@H]1[C@H](CNC1=O)c1ncn(c1)C
SN00361791	O=C1CCC(=O)Nc2c(cccc2)[C@@H]1CC(=O)c1n(cnc1)C
SN00365948	n1c2c(ccc1-c1n(cnc1)C)cccc2
SN00369505	O=C/1NC[C@@H](\C\1=C\c1ccccc1)c1ncn(c1)C
SN00380361	O1C[C@@H](Cc2ncn(c2)C)[C@H]([C@@H](O)c2ccccc2)C1=O
SN00380631	O=C/1NC[C@@H](\C\1=C/c1ccccc1)c1n(cnc1)C
SN00393484	O1[C@@]2(OC)C=C[C@]1(C)[C@H](OC(=O)\C=C\c1ncn(c1)C)C[C@H]1[C@@H](C=C2C O)[C@@H](CC=C1C)C(C)C
SN00395025	O=C1N(C[C@H]([C@@H]1[C@H](OC(=O)c1ccccc1)c1ccccc1)c1n(cnc1)C)C
SN00404046	O=C1N(C[C@H]([C@@H]1[C@H](O)c1ccccc1)c1n(cnc1)C)C
ZINC02129942	O(C)c1cc2c3N=CN(CCc4nc[nH]c4)C(=O)c3n(c2cc1)C
ZINC02133189	O=C1N(C=Nc2c1n(c1c2cc(cc1)C)C)CCc1nc[nH]c1
ZINC03984507	O=C1N(CC(=O)N2CCc3c([nH]c4c3cccc4)[C@]12C)CCn1ccnc1
ZINC03984657	Clc1cc2c3N=CN(CCCn4ccnc4)C(=O)c3[nH]c2cc1
ZINC03985037	O(C)c1cc2c3c([nH]c2cc1)[C@@]1(N(CC3)C(=O)CN(CCCn2ccnc2)C1=O)C
ZINC03985121	O1c2c(ccc(OC)c2C)C(C)=C(CCC(=O)NCCCn2ccnc2)C1=O
ZINC03985127	O1c2c(cc3c(oc(C)c3C)c2C)C(C)=C(CC(=O)NCCCn2ccnc2)C1=O
ZINC03985168	O1c2c(cc3c(oc(C)c3C)c2)C(C)=C(CC(=O)NCCCn2ccnc2)C1=O
ZINC03985169	O1c2c(ccc(OC)c2)C(C)=C(CC(=O)NCCCn2ccnc2)C1=O
ZINC03985175	O1c2c(ccc(OCC(=O)NCCCn3ccnc3)c2C)C(=CC1=O)c1ccccc1
ZINC03985184	O1c2c(c3occ(c3c(c2)C)C)C(C)=C(CC(=O)NCCCn2ccnc2)C1=O
ZINC03985196	O1c2c(cc3c4CCCCc4oc3c2)C(C)=C(CC(=O)NCCCn2ccnc2)C1=O
ZINC03985199	O1c2c(cc3CCC(Oc3c2)(C)C)C(C)=C(CC(=O)NCCCn2ccnc2)C1=O
ZINC03985204	O1c2c(c3oc(C)c(c3c(c2)C)C)C(C)=C(CC(=O)NCCCn2ccnc2)C1=O
ZINC05205207	Clc1cc2c(OC(=O)C=C2Cn2ccnc2)cc1C
ZINC06623694	O1c2c(cc3c(occ3C)c2)C(C)=C(CC(=O)NCCCn2ccnc2)C1=O
ZINC06623704	O1c2c(cc3c(occ3C)c2C)C(C)=C(CC(=O)NCCCn2ccnc2)C1=O
ZINC06624078	O1c2c(ccc(OCC(C)=C)c2C)C(C)=C(CC(=O)NCCCn2ccnc2)C1=O
ZINC06624271	O1c2c(cc3c(oc(C)c3C)c2)C(C)=C(CCC(=O)n2ccnc2)C1=O
ZINC08764507	O1c2c(cc3c(oc(C)c3-c3ccccc3)c2)C(C)=C(CCC(=O)NCCCn2ccnc2)C1=O
ZINC08789969	O1c2c(ccc(O[C@@H](C(=O)NCCCn3ccnc3)C)c2C)C(=CC1=O)c1ccccc1
ZINC08791359	O1c2c(cc3c(occ3-c3ccccc3)c2)C(C)=C(CC(=O)NCCCn2ccnc2)C1=O
ZINC08791849	O(C)c1ccccc1[C@H]1c2c([nH]c3c2cccc3)[C@@]2(N(C1)C(=O)CN(CCCn1ccnc1)C2=O)C
ZINC08791851	O(C)c1ccccc1[C@@H]1c2c([nH]c3c2cccc3)[C@@]2(N(C1)C(=O)CN(CCCn1ccnc1)C2=O)C
ZINC08791853	O1c2c(ccc(O[C@H](C(=O)NCCCn3ccnc3)C)c2C)C(=CC1=O)c1ccccc1
ZINC08791863	O1c2c(cc3c(oc4CCCCc34)c2C)C(C)=C(CC(=O)NCCCn2ccnc2)C1=O
ZINC08917761	O=C1N2[C@H](Cc3c([nH]c4c3cccc4)[C@@H]2c2ccc(cc2)C(C)C)C(=O)N(C1)CCCn1ccnc1
ZINC08917976	O(C)c1cc(ccc1)[C@H]1N2[C@@H](Cc3c1[nH]c1c3cccc1)C(=O)N(CC2=O)CCCn1ccnc1
ZINC08918526	O=C1N2[C@H](Cc3c([nH]c4c3cccc4)[C@H]2c2ccc(cc2)C(C)C)C(=O)N(C1)CCCn1ccnc1
ZINC08918535	O(C)c1cc(ccc1)[C@@H]1N2[C@@H](Cc3c1[nH]c1c3cccc1)C(=O)N(CC2=O)CCCn1ccnc1
ZINC08918541	O(C)c1ccccc1[C@H]1N2[C@@H](Cc3c1[nH]c1c3cccc1)C(=O)N(CC2=O)CCCn1ccnc1
ZINC08918542	O(C)c1ccccc1[C@@H]1N2[C@@H](Cc3c1[nH]c1c3cccc1)C(=O)N(CC2=O)CCCn1ccnc1
ZINC08964670	O=C1N2[C@@H](Cc3c([nH]c4c3cccc4)[C@H]2c2ccccc2)C(=O)N(C1)CCCn1ccnc1
ZINC08964671	O=C1N2[C@@H](Cc3c([nH]c4c3cccc4)[C@@H]2c2ccccc2)C(=O)N(C1)CCCn1ccnc1
ZINC08964674	O=C1N2[C@@H](Cc3c([nH]c4c3cccc4)[C@H]2CCc2ccccc2)C(=O)N(C1)CCCn1ccnc1
ZINC08964675	O=C1N2[C@@H](Cc3c([nH]c4c3cccc4)[C@@H]2CCc2ccccc2)C(=O)N(C1)CCCn1ccnc1

ZINC08964713	Clc1ccc(cc1)[C@@H]1N2[C@@H](Cc3c1[nH]c1c3cccc1)C(=O)N(CC2=O)CCCn1ccnc1
ZINC08964714	Clc1ccc(cc1)[C@@H]1N2[C@H](Cc3c1[nH]c1c3cccc1)C(=O)N(CC2=O)CCCn1ccnc1
ZINC08964715	Clc1ccc(cc1)[C@H]1N2[C@H](Cc3c1[nH]c1c3cccc1)C(=O)N(CC2=O)CCCn1ccnc1
ZINC11535815	O1c2c(cc(cc2)C)C(=CC1=O)Cn1ccnc1
ZINC12660802	O=C(NCCCn1ccnc1)[C@@H](CCC)C
ZINC12661337	O=C1N2[C@@H](Cc3c([nH]c4c3cccc4)C2(C)C)C(=O)N(C1)CCCn1ccnc1
ZINC12661679	O(C)c1cc2c3N=CN(CCCn4ccnc4)C(=O)c3[nH]c2cc1OC
ZINC12663441	O(C)c1cc2CCN3C(=CC(=NC3=O)NCCCn3ccnc3)c2cc1OC
ZINC12663482	O1c2c(cc(O)c(c2)-c2ccccc2)C(=CC1=O)Cn1ccnc1
ZINC12663493	O1c2c(cc(cc2)CC)C(=CC1=O)Cn1ccnc1
ZINC12663597	O1c2c(C(C)=C(CCC(=O)n3ccnc3)C1=O)c(OC)cc1OC(CCc12)(C)C

Table S2. Calculated values of pIC_{50} (2D and 3D-QSAR) and K_i (docking) and their mean [a].

ID 2	2D-QSAR	Applicability 2D-QSAR	3D-QSAR	Applicability 3D-QSAR	Docking	Mean
SN00087296	5.98	YES	6.1	Excellent	7.38	6.49
ZINC08964675	5.36	No	5.7	Good	7.80	6.29
SN00001674	7.76	YES	4.5	OK	6.49	6.25
ZINC08918535	6.08	No	5.2	Excellent	7.13	6.14
ZINC08964671	5.20	No	6.0	Good	6.86	6.02
SN00005909	5.27	YES	5.0	OK	7.76	6.01
SN00032231	5.53	No	5.5	Bad	6.92	5.98
SN00238920	4.80	No	4.9	OK	7.90	5.87
ZINC03985121	4.19	YES	6.7	Poor	6.66	5.85
ZINC08917761	6.10	No	5.2	Good	6.20	5.83
ZINC03985168	3.76	YES	5.6	OK	7.96	5.78
SN00230416	5.21	No	5.4	Good	6.53	5.71
SN00032287	4.30	YES	4.7	Excellent	8.10	5.70
SN00213775	5.42	No	5.4	Excellent	6.20	5.67
ZINC03985184	3.81	No	5.7	Good	7.44	5.65
SN00087305	4.00	No	5.3	OK	7.53	5.61
SN00032199	4.33	YES	5.4	Excellent	7.02	5.58
ZINC12663482	4.10	YES	5.1	Poor	7.39	5.53
ZINC08918526	5.35	No	5.7	Good	5.53	5.53
SN00264937	4.60	YES	5.8	Excellent	6.16	5.52
ZINC08791863	5.04	No	5.4	Poor	6.11	5.52
SN00031538	6.01	YES	5.1	Excellent	5.40	5.50
SN00032211	5.54	YES	4.7	Bad	6.26	5.50
SN00032229	4.89	YES	4.5	Bad	7.08	5.49
SN00032204	4.34	YES	4.8	Bad	7.31	5.48
SN00032224	4.59	YES	4.6	OK	7.08	5.42
ZINC03985196	4.93	No	4.8	Poor	6.53	5.42
SN00306630	4.60	No	5.6	Good	5.93	5.38
ZINC12661679	3.82	YES	4.9	Good	7.37	5.36
SN00292230	4.87	No	4.6	Good	6.62	5.36
SN00032220	4.05	YES	5.2	Poor	6.81	5.35
SN00032209	4.69	YES	4.9	OK	6.45	5.34
SN00344415	3.72	No	5.0	Excellent	7.14	5.28
ZINC06624078	4.35	YES	5.4	Good	6.08	5.28
SN00032203	4.03	YES	5.1	Bad	6.70	5.28
ZINC06624271	3.35	YES	4.4	Excellent	8.03	5.26
SN00320806	4.46	No	4.8	Excellent	6.47	5.24

MNP4778	3.78	No	5.1	Excellent	6.81	5.23
SN00032289	3.32	YES	5.1	OK	7.25	5.22
SN00032216	4.37	YES	4.9	OK	6.40	5.22
SN00032288	3.87	YES	4.7	Good	7.02	5.20
ZINC08791853	3.83	No	5.3	Bad	6.40	5.18
SN00032221	4.34	YES	4.3	Poor	6.87	5.17
SN00032205	4.12	YES	4.7	Poor	6.64	5.16
SN00065121	5.15	YES	5.1	Excellent	5.16	5.14
SN00284317	4.46	No	4.5	Excellent	6.42	5.13
ZINC03984657	2.79	No	5.7	Excellent	6.84	5.11
ZINC03985175	3.54	No	5.2	Poor	6.37	5.03
SN00032210	4.83	YES	4.9	Bad	5.31	5.01
MNP4779	2.77	No	5.1	Excellent	7.16	5.01
SN00032219	3.78	YES	5.3	Poor	5.94	5.01
SN00352003	4.30	YES	3.6	Excellent	7.10	5.00
ZINC12660802	5.25	YES	4.8	Excellent	4.95	5.00
SN00404046	4.13	No	4.2	Excellent	6.47	4.93
SN00087300	2.99	YES	5.4	Poor	6.39	4.93
SN00065009	3.48	No	4.8	Excellent	6.46	4.91
ZINC08964714	4.99	No	6.3	OK	3.40	4.90
SN00032217	3.62	YES	4.7	Good	6.34	4.89
SN00000116	4.31	YES	4.3	Excellent	5.73	4.78
SN00253274	2.97	No	4.2	Excellent	7.08	4.75
SN00361791	3.95	YES	3.5	Excellent	6.73	4.73
SN00325795	4.78	YES	3.8	Good	5.55	4.71
SN00032215	4.39	No	4.4	OK	5.32	4.70
SN00086779	4.50	YES	5.0	Excellent	4.47	4.66
MNP2902	3.72	No	4.8	Good	5.32	4.61
SN00032201	3.53	YES	4.2	Bad	6.07	4.60
ZINC08791359	3.79	No	3.9	Poor	5.95	4.55
ZINC03985127	2.93	No	4.2	Bad	6.35	4.49
SN00369505	2.51	No	4.0	Excellent	6.57	4.36
SN00261841	2.42	No	4.4	Excellent	6.01	4.28
ZINC08918542	6.66	No	5.8	Good		4.15
SN00380631	2.15	No	3.9	OK	6.10	4.05
ZINC08964674	6.11	No	6.0	Excellent		4.04
SN00243990	2.27	No	2.8	OK	6.42	3.83
SN00280678	4.89	No	6.4	Good		3.76
ZINC08964713	5.74	No	5.4	Excellent		3.71
ZINC08918541	5.91	No	5.1	Good		3.67
ZINC08917976	5.34	No	5.6	OK		3.65
SN00380361	5.78	No	5.1	Excellent		3.63
ZINC12663441	4.99	YES	5.7	OK		3.56
SN00087288	5.90	YES	4.6	Excellent		3.50
SN00032284	4.84	YES	5.6	OK		3.48
ZINC08791849	4.23	No	6.0	Excellent		3.41
SN00032225	5.18	No	4.9	Poor		3.36
SN00032212	4.94	YES	5.1	Poor		3.35
SN00393484	4.42	No	5.6	Bad		3.34
SN00265125	4.49	No	5.5	Poor		3.33
ZINC08964670	4.45	No	5.4	Good		3.28
SN00032256	4.64	YES	5.2	OK		3.28
SN00278994	5.13	No	4.7	Bad		3.28
ZINC03985199	4.66	YES	5.1	Bad		3.25

ZINC08964715	4.25	No	5.5	Excellent		3.25
ZINC08789969	4.57	No	5.0	Bad		3.19
ZINC12661337	4.85	No	4.4	Excellent		3.08
ZINC03985169	4.76	YES	4.4	Good		3.05
SN00031540	4.31	No	4.8	Poor		3.04
SN00395025	4.06	No	4.8	Excellent		2.95
SN00365948	4.30	YES	4.5	Excellent		2.93
ZINC08791851	3.49	No	5.3	Good		2.93
ZINC03984507	2.86	No	5.9	Excellent		2.92
SN00032206	3.42	YES	5.1	Bad		2.84
ZINC08764507	4.51	No	3.9	Bad		2.80
ZINC02129942	2.29	No	6.1	Excellent		2.80
ZINC03985204	3.55	No	4.8	OK		2.78
MNP4780	3.71	No	4.6	Good		2.77
ZINC12663597	3.81	No	4.5	OK		2.77
SN00257780	4.90	No	3.3	Good		2.73
SN00031541	3.51	No	4.6	Poor		2.70
SN00076641	3.78	No	4.3	Good		2.69
ZINC02133189	2.60	No	5.4	Excellent		2.67
ZINC06623704	3.20	No	4.8	Poor		2.67
SN00031537	3.60	No	4.4	Good		2.67
ZINC03985037	2.07	No	5.9	Excellent		2.66
ZINC12663493	3.05	YES	4.9	Poor		2.65
SN00031546	3.42	No	4.5	Excellent		2.64
SN00343943	3.86	No	4.0	Good		2.62
SN00031553	3.59	No	4.1	Poor		2.56
MNP5523	3.79	No	3.9	Excellent		2.56
ZINC06623694	2.68	YES	5.0	Good		2.56
ZINC11535815	2.74	YES	4.9	Excellent		2.55
SN00031548	3.43	No	4.2	Good		2.54
MNP4781	2.35	No	5.2	Excellent		2.52
SN00031547	2.94	No	4.6	OK		2.51
SN00031559	2.62	YES	4.9	OK		2.51
SN00280641	3.11	No	4.4	Excellent		2.50
SN00021256	3.79	No	3.7	Excellent		2.50
SN00335204	2.39	No	5.1	Excellent		2.50
ZINC05205207	3.39	YES	4.1	OK		2.50
SN00031551	2.88	No	4.6	Good		2.49
SN00031555	2.47	No	5.0	Poor		2.49
SN00031542	2.74	No	4.7	Excellent		2.48
SN00031564	3.23	No	4.2	Excellent		2.48
SN00031543	2.62	No	4.8	Excellent		2.47
SN00031534	2.60	No	4.8	Good		2.47
SN00031557	2.94	No	4.2	OK		2.38
SN00031563	2.73	No	4.3	OK		2.34
SN00031535	2.35	No	4.6	Excellent		2.32
SN00214383	2.55	No	4.1	Good		2.22
MNP4640	2.45	YES	4.1	OK		2.18
SN00031552	2.41	No	3.9	Excellent		2.10
SN00031561	1.96	No	4.3	Excellent		2.09
SN00031550	1.97	No	4.1	OK		2.02
MNP3090	—	—	—	—	5.43	—
SN00286074	—	—	—	—	—	—

[a] In green were highlighted the compounds possessing a calculated value of pK_i in the range 6.01–8.09 (1.00–0.01 μM).

Table S3. Calculated values of pIC_{50} (2D and 3D-QSAR) and K_i (docking) and their mean for outsider marine compounds.

ID 2	2D-QSAR	Applicability 2D-QSAR	3D-QSAR	Applicability 3D-QSAR	Docking	Mean
MNP4580	5.16	Yes	6.3	OK	7.48	6.31
MNP6510	5.94	No	5.3	Good	6.90	6.04
MNP3690	5.21	Yes	5.5	Excellent	7.11	5.94
MNP10136	4.63	Yes	5.6	Excellent	6.53	5.58
